# Spectrum and Density of Gamma and X-ray Induced Mutations in a Non-Model Rice Cultivar

**DOI:** 10.3390/plants11233232

**Published:** 2022-11-25

**Authors:** Joanna Jankowicz-Cieslak, Bernhard J. Hofinger, Luka Jarc, Sini Junttila, Bence Galik, Attila Gyenesei, Ivan L. Ingelbrecht, Bradley J. Till

**Affiliations:** 1 Plant Breeding and Genetics Laboratory, FAO/IAEA Joint Division, International Atomic Energy Agency (IAEA), 2444 Seibersdorf, Austria; 2 Bioinformatics and Scientific Computing Core, Vienna Biocenter Core Facilities GmbH, Dr-Bohr-Gasse 3, 1030 Vienna, Austria; 3 Medical Bioinformatics Centre, Turku Bioscience Centre, University of Turku, Tykistökatu 6, 20520 Turku, Finland; 4 Medical Bioinformatics Centre, Turku Bioscience Centre, Åbo Akademi University, Tykistökatu 6, 20520 Turku, Finland; 5 Department of Clinical Molecular Biology, Medical University of Bialystok, 15-269 Bialystok, Poland; 6 Bioinformatics Research Group, Genomics and Bioinformatics Core Facility Szentágothai Research Centre, University of Pécs, H-7622 Pecs, Hungary; 7 Veterinary Genetics Laboratory, University of California, Old Davis Road, Davis, CA 95616, USA

**Keywords:** mutation breeding, structural variants, forward genetics, whole-genome sequencing, transposable elements

## Abstract

Physical mutagens are a powerful tool used for genetic research and breeding for over eight decades. Yet, when compared to chemical mutagens, data sets on the effect of different mutagens and dosages on the spectrum and density of induced mutations remain lacking. To address this, we investigated the landscape of mutations induced by gamma and X-ray radiation in the most widely cultivated crop species: rice. A mutant population of a tropical upland rice, *Oryza sativa* L., was generated and propagated via self-fertilization for seven generations. Five dosages ranging from 75 Gy to 600 Gy in both X-ray and gamma-irradiated material were applied. In the process of a forward genetic screens, 11 unique rice mutant lines showing phenotypic variation were selected for mutation analysis via whole-genome sequencing. Thousands of candidate mutations were recovered in each mutant with single base substitutions being the most common, followed by small indels and structural variants. Higher dosages resulted in a higher accumulation of mutations in gamma-irradiated material, but not in X-ray-treated plants. The in vivo role of all annotated rice genes is yet to be directly investigated. The ability to induce a high density of single nucleotide and structural variants through mutagenesis will likely remain an important approach for functional genomics and breeding.

## 1. Introduction

Crop biodiversity plays a key role in overcoming existing and emerging climate-related challenges that threaten world food security. Yet, domestication and thousands of years of human selection resulted in bottlenecks that greatly reduced genetic diversity [1,2,3]. While large collections of diverse germplasm are being created and are being utilized to address food insecurity, limitations including linkage drag can hamper the timely introgression of desired traits into elite cultivars [4,5]. An alternative approach to using existing diversity for crop improvement is to generate new genetic variation. While genome editing represents the latest technological iteration, the concept is not new. Scientists continue to create novel variation in plants as they have since the 1920s [6]. The first mutagenic treatments were performed by irradiating cells with X-rays. Pioneering work was carried out in the insect *Drosophila melanogaster* and shortly thereafter in plants [7,8]. X-ray irradiation is an important tool for genetic research. In *D. melanogaster* and *C. elegans*, X-ray irradiation was used to create balancer chromosomes that facilitated stock management and continue to be used as a tool for genome research [9,10]. In crops, the first “mutant” variety was created using X-ray irradiation of tobacco and released in the 1930s [11]. In the decades following, gamma irradiation became the predominant method for crop mutation breeding, with the earliest variety found in the IAEA’s Mutant Variety Database being the “Pink Hat” rose released in 1960 (https://mvd.iaea.org/, accessed 15 September 2022). Today there are more than 3360 officially registered mutant varieties in the IAEA’s Mutant Variety Database, with approximately 50% of varieties developed from direct or indirect use of gamma- and 17% from X-ray irradiation. This compares to approximately 11% of varieties derived from chemical mutagenesis and less than 1% listed as being derived from ion beam irradiation (https://mvd.iaea.org/, accessed 15 September 2022). The exact number of unique mutation events that contributed to varieties is difficult to estimate owing to the fact that some founder varieties were used to introgress traits in other backgrounds [12]. However, the total value of mutant varieties is estimated to be in the billions of dollars, showing the effectiveness of random mutagenesis in crop improvement [13].

The causative mutations leading to improved mutant varieties and their underlying mechanisms remain largely unknown. In addition to furthering our understanding of gene function, knowledge of the effect of mutagen and dosage on the number and type of heritable induced mutations will facilitate optimizing the mutation breeding projects so that the maximum probability of achieving the desired trait can be obtained with the smallest population size. Of the more than 240 species where mutations were used to create new varieties, rice (*Oryza sativa* L.) is the most prominent, representing approximately 25% of all registered varieties (https://mvd.iaea.org/, accessed 15 September 2022) [14]. Indeed, rice is a staple for more than half of the world’s population and a future with sustainable food security must include approaches to make rice more climate resilient [5].

The availability of relatively low-cost DNA sequencing enabled a genome-wide view of the effect of mutagens on plant genomes. Large data sets exist for chemically mutagenized plants. Decades of research using the chemical mutagen ethyl methane sulfonate (EMS) revealed that the mutagen produces primarily G:C to A:T point mutations with limited positional bias [15,16,17]. The effect of physical mutagens on plant genomes is more complex and less clear. Whole-genome sequencing of 1504 rice mutants (variety Kitaake) treated with fast neutron mutagenesis revealed a broad spectrum of induced mutations, including insertions, duplications, and single-base substitutions [18]. Whole-genome sequencing of seven rice mutants (variety Hitomebore) treated with C-ion and seven treated with gamma-rays showed both mutagens producing single-base substitutions, indels, and larger structural variations with more structural variants recovered in C-ion mutants [19]. An additional study of six gamma-irradiated lines (cultivar Nipponbare), also showed a predominance of single-base substitutions with indels and structural variants accumulating at a lower frequency [20]. More recently, a larger-scale study of 123 gamma-irradiated rice (subsp. japonica cv.) mutant lines created for TILLING assays focused on the recovery of single-base and small indel mutations and found a higher percentage of indels compared to SNVs [21]. Larger insertion, deletion and structural variations were not evaluated in this study. In contrast, while numerous reports were published on the effect of X-ray irradiation on rice phenotype, there is limited information on the effect of X-ray treatment at the sequence level in plants. When considering mutation breeding, a bulk of the seeds from a single cultivar are typically irradiated. In addition, mutation breeding often involves the choice of locally adapted cultivars where information is limited on the effect that genotype may have on the accumulation of induced mutations.

To address the limited knowledge of the effect of X-rays on the rice genome, and to expand knowledge on gamma irradiation, and the spectrum and density of induced mutations in a different rice cultivar, mutant populations of a Malagasy rice variety were developed and evaluated. The effect of dosage showed gamma-irradiated material to have more survivability over a broader range. The highest density of mutations (8816) was observed in the highest dosage (450 Gy). This trend was not observed in X-ray-irradiated material where one of four mutant lines treated with 75 Gy had 3-fold more accumulated mutations than the mutant with the lowest number at the same dosage.

## 2. Results

### 2.1. Generation of Mutant Population and Mutant Selection

**Figure 1:**
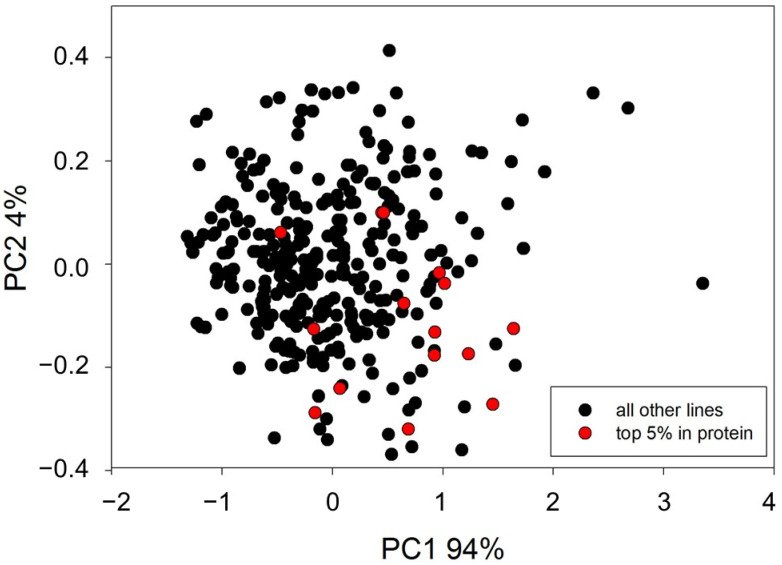
PCA of NIRS data on 329 mutant rice lines. Lines with seed protein in the top 5% of the total are marked in red.

**Figure 2:**
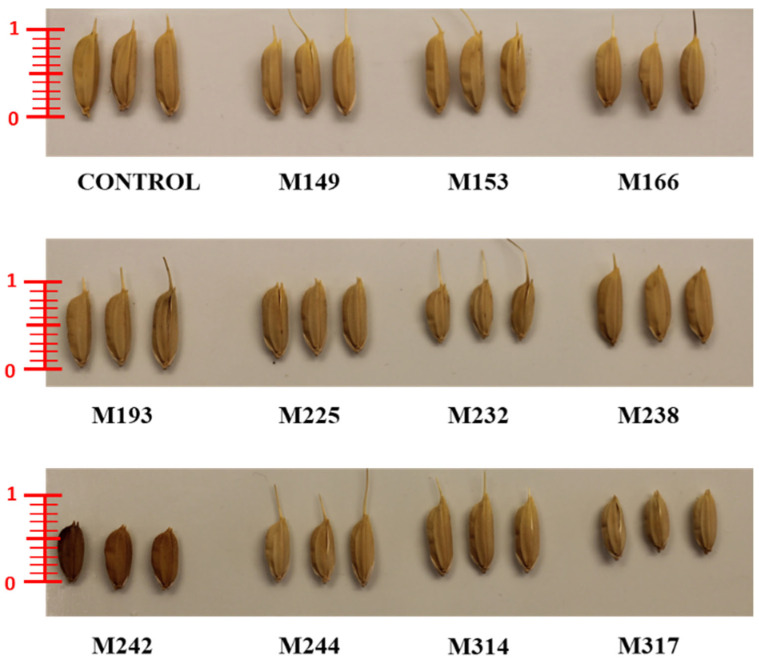
Grain length and color variation in rice mutant lines (scale used cm).

To evaluate the effect of gamma and X-ray irradiation on *Oryza sativa* L. ‘Marotia’, seeds were treated with one of six selected dosages of gamma irradiation or one of six dosages of X-ray irradiation. The survival rate of M_1_ plants negatively correlated with dosage starting at 150 Gy (Table 1). Prior to DNA sequencing, plants were evaluated for phenotypic variation with the rational that variation in plant phenotype may indicate the presence of novel nucleotide variation. Phenotypic variation between mutated and non-mutated control material was observed for survival rate, flowering date, plant height, panicle length, and number of seed per plant (Table 2). In addition, a subset of 329 mutant lines were subjected to qualitative and quantitative near-infrared reflectance spectroscopy (NIRS), with data collected for ash, fat, fibre, protein, and moisture. Principle component analysis (PCA) resulted in a clustering of samples with 14 lines as statistical outliers within two or more standard deviations from the mean (Figure 1). While the small sample size used in NIRS screening did not uncover mutants with higher seed protein content, the variation observed in NIRS and the other phenotypic traits measured suggested that genome sequencing may uncover novel induced mutations. Data from phenotypic analyses was therefore. used to select 11 mutant lines for genotypic evaluation. (Table 2 and Figure 2).

### 2.2. Genome Sequencing

Sequencing was performed using an Illumina HiSeq2500 system and 2 × 125 PE reads on genomic DNA from two biological replicates of each of the 11 selected mutants and non-mutated control. Between 125,509,494 and 182,922,162 reads were produced, resulting in a mean coverage between 26.4 and 37.0 for all samples sequenced (Supplemental Tables S1 and S2, Supplemental Figure S1).

### 2.3. Single Nucleotide and Insertion/Deletion Variants Detected in Rice Mutant Lines

SNV and small indel variants were identified using GATK HaplotypeCaller. Variants found in the non-mutagenized control material, variants previously identified by resequencing non-mutagenized plants, and variants common in more than one mutant line were considered natural variations and not reported as induced mutations (Supplemental Table S3). This resulted in between 18,612 and 6069 total mutations per plant, with more than 90% being SNV and small indels in all irradiated material. This represents an estimated mutation frequency between 1 mutation/23 kb and 1/71 kb (Table 3). No clear trend between dosage and mutation frequency was observed. Line M242 (treated with 75 Gy X-rays) had the highest number of SNV and indel variants. Other lines treated with 75 Gy showed slightly higher accumulation of indels compared to 150 Gy X-ray-irradiated lines, with SNV variants sometimes higher and sometimes lower. In gamma-irradiated material, plants from 150 Gy-irradiated seeds accumulated more SNVs than 300 Gy gamma-irradiated material and more indel mutations than either 300 or 450 Gy gamma-irradiated material.

### 2.4. Structural Variants Detected in Rice Mutant Lines

**Figure 3:**
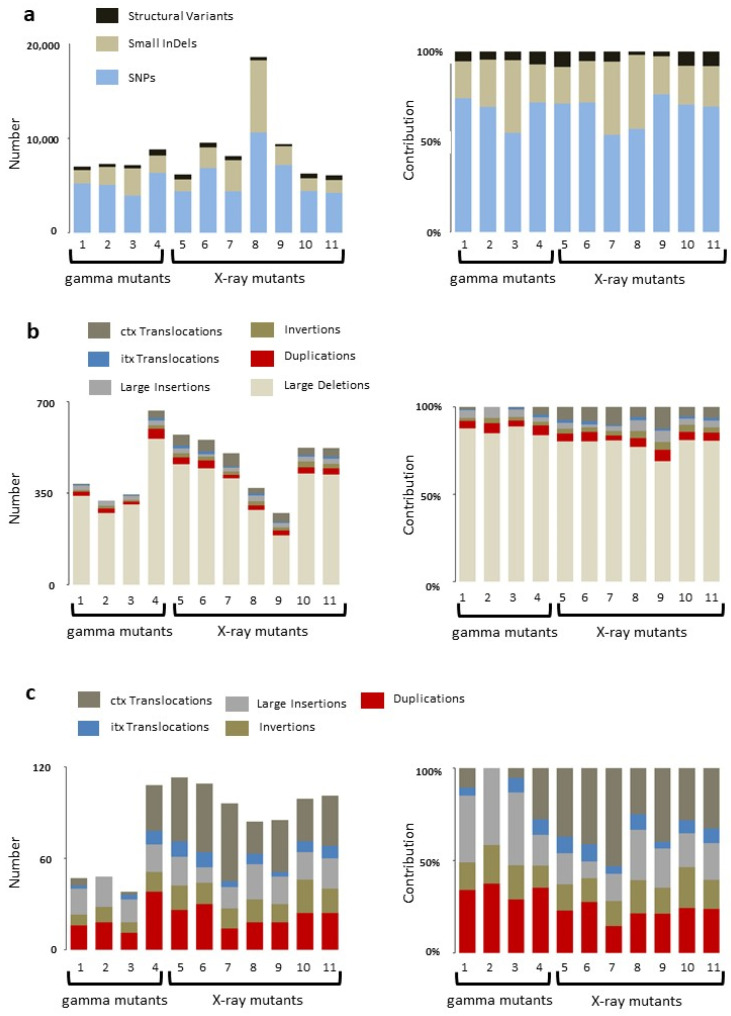
Distribution of variants in mutant lines: (**a**) total variation identified in gamma and X-ray mutagenized rice; (**b**) distribution of SVs; and (**c**) comparison of the least represented SVs in rice mutants.

Discovery of larger structural variants (SVs) was carried out using the programs Manta, Lumpy, Breakdancer, and bin-by-sam. Large deletions, insertions, inversions, duplications, and translocations unique to mutant lines were identified. A comparison of SNV and indel variations versus SVs showed that at least over 90% of induced mutations are SNVs and indels with structural variants making up between 1.8% and 8.5% of total variation (Table 3 and Figure 3a). The largest number of structural variants was identified in the highest (450 Gy) dosage gamma-irradiated line, with the second largest recovered in a line treated with the lowest dosage of X-rays (75 Gy). Evaluation of structural variants revealed large deletions to be the predominant SV, comprising more than 80% of all SVs (Figure 3b). The remaining SV types were present at varying ratios in mutants with intra-chromosomal translocations (itx) being the least common (Figure 3c). While the overall number of SV events is low, the percentage of the genome affected is high. For example, evaluation of mutant M149 revealed 340 deletion events covering 49.5 Mbp (Supplemental Table S4). Of these, 126 (37%) are within intergenic regions, and 213 (63%) span genes with 141 deletions (42%) spanning regions annotated as containing transposons or retrotransposons.

Interestingly, the total number of translocations predicted within X-ray-irradiated material is higher than that identified in gamma treated samples, with less of an observable trend based on dosage (Supplemental Figures S2 and S3). A large number of translocations are also predicted between non-mutagenized parental genotype and the reference genome (Supplemental Figure S3). Intrachromosomal translocations are predicted at a lower frequency in mutated material.

### 2.5. Validation of Predicted Mutations

Twenty-four small variants (SNVs and short indels) unique to a single mutant line and six non-unique variants were selected for validation by Sanger sequencing. To test for the possibility of false negative errors caused by true mutations being removed during the data filtering step, four putative variants that did not pass filtering parameters were also sequenced. Three of the four were removed from the data because of the allele frequency threshold, and one predicted variant was removed due to low coverage. All 30 variants predicted by GATK and passing downstream filtration were confirmed by Sanger sequencing. None of the GATK-predicted variants removed due to allele ratio or coverage could be identified by Sanger sequencing (Table 4). In addition, 27 predicted larger structural variants ranging in size from 179 to 7732 were all confirmed by PCR size polymorphism (Table 5).

### 2.6. Predicted Effect of Induced Mutations

The potential effect of SNV and indel mutations on gene function was evaluated using SNPeff. The frequency of nonsense changes ranged between 0.38 and 1.99% (Table 6). Similarly, high impact SNVs represented the lowest frequency with the majority of variation found in intergenic regions. The distribution of indels is similarly highest in intergenic regions. In contrast to SNVs, predicted high-impact indels predominate over low and moderate ones (Table 6). Larger variants were also recovered that affect coding regions (Table 5).

## 3. Discussion

When inducing novel mutations, a balance must be struck whereby a sufficient type and number of variants accumulate that are transmissible to the next generation, while at the same time limiting plant death and sterility. Phenotypic measurements, such as hypocotyl length and survivability, are typically conducted in the first (M_1_) generation to estimate the effect of mutagen dosage [19]. When using seed mutagenesis, the first generation is chimeric, making the link between early observed phenotypes and heritable mutations difficult, as plants undergo a broad response to irradiation-induced DNA damage that includes cell cycle arrest [22]. In the current study, survival rates increased slightly at lower dosages compared to control (0–150 Gy for gamma and 0–75 Gy in X-ray) and then dropped as dosage increased. While small variations may represent experimental stochasticity, low dosages of ionizing radiation are reported to have a stimulating effect on plant growth, a process known as hormesis [23]. Further studies are required to evaluate if low dosage irradiation has an effect on seed germination and plant growth.

Genome sequencing of rice and other species revealed that gamma irradiation induces a broad spectrum of heritable mutations ranging from single nucleotide variants to large chromosomal aberrations [19,24,25]. In *Arabidopsis thaliana*, studies were undertaken to evaluate the transmissibility of mutations in gamma and carbon ion-irradiated pollen, suggesting a link between non-transmissible large deletions and semisterility [26]. This phenomenon is likely also occurring in plants where seed is mutagenized. In the current study, increasing gamma irradiation dosage from 150 Gy to 600 Gy resulted in survival rates dropping from 80% to 2%. The highest dosage evaluated at the DNA sequence level, 450 Gy, resulted in the highest accumulation of mutations (8816), including the highest overall number of structural variants (637). This represents 19% more mutations than 300 Gy treated material, but also a drop in survivability from 76% to 28%. In rice seed (cv. Nipponbare) treated with gamma irradiation (a 137 Cs source versus 60 Co used in the current study) more mutations were reported in lower dosages (4698 at 165 Gy) compared to the highest dosage (3326 at 389 Gy). In addition, the highest dosage produced the lowest number of structural variants [20]. This is in contrast with the present study, where the number of structural variants were highest in 450 Gy-treated material, followed by the next highest in plants from 150 Gy-treated seed. These differences likely occur due to a combination of variation in treatment conditions such as seed moisture content, source of gamma rays, and also genotype-specific DNA and epigenomic variation, and differences in other chromosome features that are shown to affect spontaneous mutations in plants [27,28]. In addition, care must be taken when interpreting results, as the number of mutant lines subjected to whole-genome sequencing can be low, and it may be difficult to remove all natural or spontaneous mutations prior to estimating the frequency of induced mutations. For example, Li et al. sequenced six gamma-irradiated plants and found the total number of SNV and small indel mutations to range between 3135 and 4698 [20]. In the present study, four lines selected based on high phenotypic variability showed a range of between 8816 and 7016. When considering that random mutagenesis might produce a distribution of mutation frequencies in a collection of treated seed, such observed differences may be explained as a sampling bias in the present study towards highly mutagenized material. However, while efforts were made to remove natural genetic variation and Sanger sequencing revealed no false positive or false negative errors, an inflation of mutation frequency due to the presence of natural variants cannot be ruled out in the current data set. Bulk seed was chosen for irradiation to mimic the standard practice used in mutation breeding. The observed frequency of small induced mutations (1/23 kb to 1/71 kb) is much higher than mutation rates reported in rice (~1/135 kb) and more similar to rates reported in diploid, tetraploid, and hexaploid wheat (1/92 kb, 1/51 kb, and 1/24 kb, respectively) [6]. To control for natural variation, each biological replicate from the same mutant line was compared to two non-irradiated controls and 20 other plants from different mutant lines, and only mutations unique to the mutant line and also present in both replicates were considered induced mutations. The level of segregation distortion at the sequence level is unknown for *Oryza sativa* L. ‘Marotia’, and therefore it remains possible that sequencing of more plants from the same bulk seed is required to ensure removal of all potential natural variants present in the population. In addition, spontaneous mutations occurring during the propagation of mutated plants would be indistinguishable from irradiation-induced mutations. Studies in Arabidopsis suggest that plants may experience increased rates of spontaneous mutations after multigenerational growth in elevated temperatures [29]. For example, in mutation accumulation populations grown in high heat, a mean of 36.6 total novel SNV and indel spontaneous mutations were reported. This represents less than 1% of predicted induced mutations of the same class in any of the mutant lines described in the present study. Less is known about sequence variation due to X-ray irradiation in plants [30]. In the present study, the survival rate peaked at 82% in 75 Gy-treated material with a reduction to 70% in 150 Gy, followed by a sharp drop off to 6% at 300 Gy. Total mutation accumulation was highly variable with a threefold variation observed at 75 Gy. Interestingly, the ratio of structural variants to total mutations was highest in X-ray-irradiated material with the lowest number of total accumulated mutations. In addition, more translocations were predicted within X-ray-irradiated material. This may indicate an increase in the generation of double strand breaks, variation in response of the DNA repair machinery, or a combination of both [31]. Whole-genome sequencing of many more samples will be required to determine the extent to which dosage and environmental conditions influence the spectrum and density of induced mutations in both gamma and X-ray-irradiated material. This will become more amenable as sequencing prices continue to drop.

Naturally occurring structural variation was implicated in plant phenotype variation, adaptation, and domestication [32,33]. In rice, a tandem duplication of the GL7 locus was shown to lead to an increase in grain length [34]. Large-scale structural variant analysis in 3000 genomes resulted in the discovery of 63 million variants, suggesting an important role for natural SVs in gene function and phenotypic diversity in rice [35]. Thus, it is expected that mutation-induced structural variants will have a large impact on plant phenotype. Indeed, this may explain the popularity of physical irradiation compared to chemical mutagenesis in plant mutation breeding. Large single-loci events are easier to genetically fix and maintain in a population as compared to traits that require multiple small mutations in unlinked genomic regions. More studies are needed to understand the mechanisms of SV accumulation in mutated plants. For example, transposons were implicated as drivers of plant genome plasticity and can function synergistically with DNA repair mechanisms to generate structural variation [36]. Irradiation of plant cells induces double strand breaks, affects DNA methylation state, which can change transposon activity, and activates DNA repair machinery [37,38]. Consequently, numerous processes, along with plant genotype, can contribute to the accumulation of germ-line mutations, making a priori predictions regarding spectrum and density of mutations from different irradiation dosages difficult.

The use of chemical mutagens or ionizing radiation can produce thousands of novel induced mutations per mutant line. This was exploited for high-throughput reverse genetic screens, such as TILLING [6]. While precision genome editing tools were developed since the advent of TILLING, new screening approaches promise continued value for reverse genetics with random mutagenesis. Automated phenomics can more efficiently and accurately link genotype to phenotype.

In addition, by combining high-density mutagenesis and large population sizes, DNA libraries can be prepared where there is a high probability of novel mutations at all base pair positions that are the target of the applied mutagen. This allows the application of genotypic screens for a specific desired base pair change, rather than discovery of all mutations within a PCR amplicon that is common for typical TILLING by sequencing screens. Genotypic screening can provide increased throughput at reduced costs. A proof of principle of this approach was described by Knudsen et al. who used digital PCR to identify EMS-induced mutations in barley in a method known as fast identification of nucleotide variants by DigITal PCR (FIND-IT) [39]. Thus, mutant populations can approach the precision of genome editing. Nevertheless, when utilizing a mutagen that produces thousands of heritable mutations per line, causative versus background mutations must be considered. Successive rounds of self-fertilization and single-seed descent can be used to fix a desired trait without genotypic evaluation so long as background mutations do not have a pleiotropic effect. Indeed, of the 872 mutant rice varieties listed in the Mutant Variety Database, 462 (53%) are listed as being directly released without crossing to another genotype. Alternatively, the desired trait can be introgressed into an elite line. This can be advantageous, as background mutations may reduce fitness. Mutation load can be reduced through repeated rounds of backcrossing or through applying genomic background selection where a small number of molecular markers can be used to select progeny with a higher percentage of the elite genome [12]. In the present study, phenotypic variation in mutants was observed throughout several generations in a number of traits, including fat, fibre, and protein content in seed, seed morphology, days to flowering, and 1000 seed weight. The extent to which these are controlled by single or multiple genes is unknown. The high percentage of direct-release rice varieties may reflect a combination of the ease of fixing mutations in self-fertile plants and the need to maintain mutations in multiple loci for the expression of the desired trait (s). Tools to rapidly map mutations can be used to address the gap in knowledge regarding the causative genes involved in many economically important mutant varieties [40].

While next generation sequencing became more routine, challenges still exist for the discovery and analysis of induced mutations using short-read sequencing. This includes the accurate assignment of heterozygous SNV mutation calls. Evaluation of carbon ion and gamma-ray-induced mutations in rice showed a high correlation between the variant allele frequency and genotype call accuracy when using GATK Haplotype caller [19]. In the current study, applying an 80% ratio to support homozygous calls allowed accurate recovery of heterozygous SNVs when evaluated using Sanger sequencing. Recovery of larger variants from short-read data is more challenging, as the estimating extent of false negative errors from different tools remains difficult. Use of longer-read sequencing will enable a more comprehensive view of mutation-induced structural variants in plants. Indeed, genomic tools are allowing a deeper understanding of plant genome plasticity. The rate of spontaneous mutations was reported to vary between different tissues [41]. In the context of mutation breeding, this highlights the utility of including biological or technical replicates so that spontaneous mutations can be differentiated from induced mutations.

Researchers and plant breeders now have a powerful suite of tools to understand gene function and improve crops as compared to 80 years ago when inducing mutations in a plant was first described. While targeted genome editing approaches, such as CRISPR, promise to revolutionize agriculture, the in vivo function of the majority of annotated plant genes remains yet to be established. This limits target selection in reverse genetic methods. Thus, it is expected that random mutagenesis and forward genetics will remain a useful approach for establishing gene function, and also for crop breeding.

## 4. Materials and Methods

### 4.1. Plant Material and Generation of a Mutant Population

An upland, local rice cultivar, Marotia (synonym CNA4136, collection number 3729), was obtained from the Antananarivo University, Antananarivo, Madagascar. Reported phenotypic characteristics are a growth cycle of 115–120 days, plant height of 120–130 cm, semi-erect plant architecture, and semi-long seed (paddy length 9.6 mm, caryopsis length 7.2 mm) with the 1000 seed weight of 33.1 g. The cultivar is characterized as lodging-sensitive. A bulk of 600 grains was mutagenized with gamma or X-rays at 0, 75, 150, 300, 450, and 600 grays of irradiation using Co-60 source located at the Plant Breeding and Genetics Laboratory in Seibersdorf, Austria (https://www.iaea.org/topics/plant-breeding/laboratory, (accessed 15 September 2022)). Seeds were pre-germinated in petri plates and upon germination transplanted into a hydroponic growth system. Survivability was determined by evaluating 50 seeds per dose per treatment type (X-ray or gamma irradiation), and carried out according to [27]. Cultures were maintained following procedures described in [42]. Non-irradiated seeds served as control material during the screening process. Upon flowering, every M_1_ panicle was bagged in order to avoid cross-contamination and to ensure a pureness of the resulting line. Over 4600 M_2_ seeds were harvested from mutagenized material. Harvested seeds were labeled following the designed nomenclatures reflecting the origin of the seed, irradiation mode, and dose applied. This nomenclature was maintained over the next propagation cycles with the addition of information on the mutant generation. The mutant population was maintained and multiplied following the principle of single-panicle descent by self-fertilization to the seventh generation (M_7_) [27]. Plants were maintained in the greenhouse with the temperature set to 28 ± 3 °C and humidity to 80 ± 5%. From November to April, artificial lights were supplemented to maintain the light intensity and the day/night period to 14/10.

### 4.2. Glasshouse Trials and Agronomic Traits Measurement for Forward Genetics

**Figure 4:**
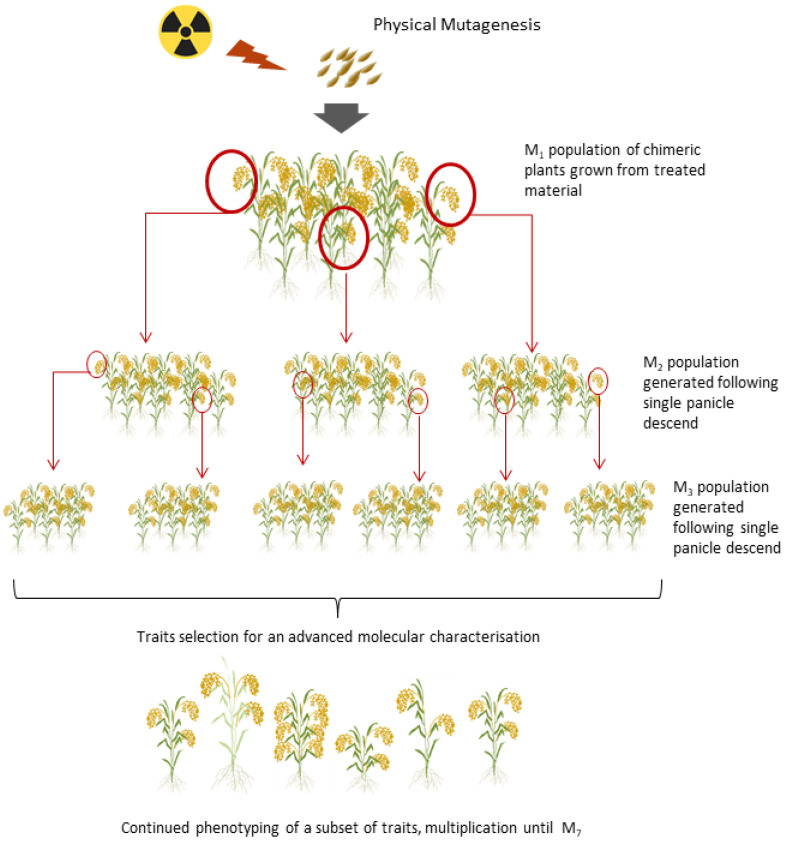
A mutant population was generated with the use of gamma or X-ray at different doses covering the range between 75 and 600 Gy plus a 0 Gy control. Plants were grown in glasshouse conditions with a hydroponic system. Single panicle descent was used for maintenance of mutant lines and for subsequent mutant selection.

All glasshouse experiments were performed in hydroponic systems using a randomized complete block design [27]. Each mutant line was grown with a planting density of 4 cm × 4 cm using the test platforms as described in Bado et al. [43]. As of M_2_ (Figure 4, Supplemental Figure S4), agronomic traits were measured for every single plant. These included flowering date, plant height, panicle length, number of panicles per plant, number of tillers per plant, number of empty and fertile spikelets, 1000-grain weight, and total number of seed. Phenotypic characterization was repeated for every mutant generation until the M_4_, at which stage 329 independent lines were identified for near infrared reflectance spectroscopy (NIRS) [44]. Seed weight and seed number are important considerations when selecting the generation to screen with the use of NIRS; therefore, obtained yield was the main criterion in the identification of lines to be analyzed.

Qualitative and quantitative NIRS analyses were performed as outlined in Vollmann and Jankowicz-Cieslak [44]. Spectral data obtained during measurements were subjected to principal component analysis (PCA). PCA scores for samples were calculated and further used in score plots to visualize classification results. In the rice mutant population, spectroscopic outliers were subsequently detected based on the distance to untreated control genotypes of the same genetic background. For quantitative analyses, external calibration was used for prediction of analyte values, which availed the measurement of traits such as ash, fat, fibre, protein, and moisture content in the subset of 329 rice mutant lines.

### 4.3. DNA Sequencing and Read Mapping

Five seeds per selected M_7_ mutant line and a control were planted (germination rate was 100%). All germinated seedlings were transplanted into a hydroponic system and allowed to grow until a second flag leaf appeared. Two healthy seedlings per line and two from the control were randomly selected for tissue collection. Young leaf tissue was harvested into Eppendorf tubes, frozen in liquid nitrogen, and stored in a −80 °C freezer until further use. High-quality genomic DNA was isolated using the Qiagen DNeasy kit (Qiagen, Hilden, Germany) following the manufacturer’s instructions. To determine stock concentrations of each sample, DNA was quantified using a NanoDrop spectrophotometer (Thermo Scientific, Waltham, MA, USA) and Qubit^®^ 2.0 Fluorimeter (Qubit^TM^ Assays, Invitrogen, Waltham, MA, USA) following manufacturer’s instructions. DNA was then diluted to the working concentration of 1 ng/µL in the final volume of 100 µL. In order to produce sequencing libraries, 60 ng of each DNA in 50 µL volume was fragmented in a microTUBE AFA Fiber (Pre-Slit-Snap-Cap 6 × 16 mm; Covaris, Brighton, UK) to a size ranging of 550 bp using Covaris M220 Focused ultrasonicator (Covaris, Brighton, UK). Fragmented, double-stranded DNA was quality checked with the use of a Bioanalyzer instrument (Agilent, Santa Clara, CA, USA) and libraries were prepared using the NEB DNA Ultra kit. The libraries were sequenced on an Illumina HiSeq2500 instrument using paired-end mode and 125 bp read length. Sequencing was targeted to a sequencing depth of 30-fold for each rice sample. The quality of the raw sequence reads was analyzed with FastQC [45]. The 2 × 125 bp paired-end sequence reads were mapped to the Oryza sativa subsp. japonica reference genome (IRGSP 1.0.23 build) using the mapping tool Burrows–Wheeler Aligner-MEM (version 0.7.17-r1188) [46]. Coverage analysis was carried out on BAM files using SAMtools depth [47].

### 4.4. Detection and Analysis of Point Mutations and Small Indels

Variant calling for SNVs and small indels was performed with GATK HaplotypeCaller (Version 4.1.8.1, Broad Institute, Cambridge, MA, USA) using default settings and following best practices, including marking and removing duplicates (Picard MarkDuplicates), and producing a multi-sample genomic variant call format file (gVCF) [48]. The multi-sample gVCF was split by sample and filtered for a minimum coverage depth of 20× using SAMtools. Zygosity calls were recalibrated such that heterozygous calls with less than 20% or more than 80% of reads supporting the alternative allele were re-scored as homozygous reference or homozygous alternative, respectively, based on previous work studying the effect of allele ratios and false positive errors in mutagenized rice [19]. Identification of variants unique to a specific mutant line was carried out using pairwise analysis with bcftools isec (version 1.10.2, Sanger Institute, Hinxton, England, Broad Institute, Cambridge, Massachusetts, United States of America) [47]. Four filtering steps were applied to remove natural nucleotide variation not induced by irradiation. First, variants were cataloged from 2 non-irradiated plants grown from the bulk seed for mutagenesis, with the first (#43801) having 1,810,784 variants and the second (#43802) having 1,814,721, representing 2,147,350 unique variants. When present, these variants were removed from the mutated material (see Supplemental Table S3). Second, variation present in more than one mutant line was considered to be natural and was removed. Third, variation not present in both biological replicates from the same line was removed. Finally, putative natural nucleic variation present in the SNP-Seek database from resequencing 3000 rice genomes found in the mutant lines was also removed [49]. The effect of the remaining small, predicted induced mutations was evaluated using SNPeff (version 5.0e, Wayne State University; Detroit, Michigan, United States of America; McGill University; Quebec, QC, Canada) [50]. A subset of called variants were selected for Sanger sequencing validation, including variants specific to a single mutant line, those common to more than one line, and also negative controls consisting of called variants that were filtered from the data set due to low coverage or allele ratio (see Table 5). Primer pairs were designed using the online tool ‘Primer3′ with the following parameters; primer size: 18–24 (opt: 22) and primer Tm 58–62 (opt: 60) [51].

### 4.5. Genomic Variant Detection: Structural Variants

BreakDancer [52], Lumpy [53], bin-by-sam [54], and Manta [55] were used to detect structural variants (SVs). Parameters for BreakDancer (version 1.1.2, Washin1gton University School of Medicine, St. Louis, MO, USA) were the default—except for −r 50—minimum number of read pairs required to establish a connection. Only translocations, which were supported by more than 100 reads, were used for further analysis. Overlapping translocations within sample replicates were merged into one if they shared similar start and end coordinates (±2000 bp) in the targeted chromosome. Default parameters were used for Manta (version 0.2.13, Illumina, San Diego, CA, USA). For Lumpy (version 1.0.2, University of Virginia, Charlottesville, VA, USA) default parameters were used, followed by filtering for the number of reads supporting one SV using both 10 and 50 reads. Bin sizes of 1, 5, 10, and 100 kb were used in bin-by-sam (version 2.0, University of California, Davis, CA, USA) analysis. The effect of the resulting structural variants unique to a specific mutant line were annotated using intansv [36].

To validate candidate SVs, regions with a large (>2 kbp), medium (~1 kbp), and smaller (<1 kbp) deletion were chosen for analysis. Each region was visualized using IGV and PCR primers designed to flanking sequence (Supplemental Figure S4). Depending on the size of the region and the extent of the deletion in the region, primers were designed for PCR products of size ranging between 526 and 7932 bp. Primer pairs were designed using the online tool ‘Primer3′ using the same parameters as for Sanger sequencing validation. To detect the presence/absence of molecular weight variations due to insertions or deletions, 5 µL of the PCR product and 2µL of orange G loading dye were loaded into wells on a 1.5% agarose gel with ethidium bromide.

## Figures and Tables

**Table 1:** Survival rates (%) of irradiated material after one month of hydroponic growth.

Treatment	0 Gy	75 Gy	150 Gy	300 Gy	450 Gy	600 Gy
gamma	74	78	80	76	28	2
X-ray	72	82	70	6	0	0

**Table 2:** Phenotypic characteristics of 11 mutant lines and the control selected for sequencing.

Sample	Treatment	Days to Flowering	Plant Height	Panicle Length	Total No of Spikelets	Empty Spikelets	Fertile Spikelets	1000 Seed Weight
Control	N/A	117	80	8	26	18	8	27.19
M149	gamma 150 Gy	90	112	14	67	28	40	30.07
M153	gamma 150 Gy	97	125	23	202	75	127	33.43
M166	gamma 300 Gy	103	100	16	49	20	30	31.94
M193	gamma 450 Gy	94	120	14	68	26	42	30.8
M225	X-ray 75 Gy	107	75	13	38	18	20	25.52
M232	X-ray 75 Gy	90	72	10	45	19	26	19.64
M238	X-ray 75 Gy	87	105	17	102	25	76	29.64
M242	X-ray 75 Gy	108	52	6	19	8	11	21.35
M244	X-ray 75 Gy	101	75	13	50	26	24	20.94
M314	X-ray 150 Gy	97	80	12	38	17	21	26.22
M317	X-ray 150 Gy	89	80	10	25	9	16	24.49

**Table 3:** Distribution of all predicted induced mutations identified in mutant rice.

Mutant Line	Treatment Type	Total Mutations *	SNVs	Indels	Total SNV/InDel Mutations	SNV/indel Mutation Frequency (bp/Event) **	Mutation Frequency All Variants bp/Event) **	SVs	Large Deletions	Duplications	Inversions	Insertions	Itx ***	Ctx ***	SV Frequency (bp/Event) **
43,801 (control) *	None	0	0	0	0	0	0	0	0	0	0	0	0	0	0
43,802_control	None	0	0	0	0	0	0	0	0	0	0	0	0	0	0
M149	gamma 150 Gy	7016	5205	1432	6637	64,788.31	61,288.48	379	339	16	7	17	2	5	1,134,564.64
M153	gamma 150 Gy	7294	5054	1919	6973	61,666.43	58,952.56	321	273	18	10	20	0	0	1,339,563.86
M166	gamma 300 Gy	7147	3924	2884	6808	63,160.99	60,165.10	339	306	11	7	15	3	2	1,268,436.58
M193	gamma 450 Gy	8816	6328	1861	8189	52,509.46	48,774.95	627	558	38	13	18	9	30	685,805.42
M225	X-ray 75 Gy	6158	4389	1248	5637	76,281.71	69,827.87	521	460	26	16	19	10	42	825,335.89
M232	X-ray 75 Gy	9546	6859	2188	9047	47,529.57	45,045.05	499	445	30	14	10	10	45	861,723.45
M238	X-ray 75 Gy	8128	4378	3303	7681	55,982.29	52,903.54	447	406	14	13	14	4	51	961,968.68
M242	X-ray 75 Gy	18,612	10,619	7652	18,271	23,534.56	23,103.37	341	285	18	15	23	7	21	1,260,997.07
M244	X-ray 75 Gy	9392	7172	1984	9156	46,963.74	45,783.65	236	188	18	12	18	3	34	1,822,033.90
M314	X-ray 150 Gy	6271	4425	1357	5782	74,368.73	68,569.61	489	425	24	22	18	7	28	879,345.60
M317	X-ray 150 Gy	6069	4216	1372	5588	76,950.61	70,851.87	481	421	24	16	20	8	33	893,970.89

* Nucleotide variation identified in non-mutated control material is considered to be natural variation. Natural variants found in mutated material are removed prior to mutation counting. ** Mutation frequency calculated as (genome size = 430 Mbp)/# observed mutations. *** Translocations are also recorded as deletions from the original location. This is reflected in the total SV count.

**Table 4:** Validation of variants by Sanger sequencing.

Mutant Line	Mutation Type	Mutation Name	Position Call	Mutation	Type	Sanger Confirmed
M149	indel	149_IND1	1:23,789,397	GAT to G	Unique in mutant	Y
M153	indel	153_IND4	4:15,522,985	C to CTCAGCCG	Unique in mutant	Y
M166	indel	166_IND1	1:18,131,046	AGCGCATGTGCCATG to A	Unique in mutant	Y
M166	indel	166_IND3	6:5,694,121	CA to C	Unique in mutant	Y
M193	SNP	193_SNP1	2:32,736,082	A to ACCAACG	Unique in mutant	Y
M193	SNP	193_SNP4	6:28,952,090	A to G	Unique in mutant	Y
M193	SNP	193_SNP5	7:10,033,642	T to C	Unique in mutant	Y
M193	SNP	193_SNP6	12:24,819,926	C to A	Filtered for allele ratio *	N
M193	indel	193_IND6	12:1,851,681 Identified in IRRI set	AC to A	Unique in mutant	Y
M225	SNP	225_SNP2	3:30,121,299	T to G	Unique in mutant	Y
M225	SNP	225_SNP3	5:18,773,517	G to A	Not unique in mutant	Y
M232	indel	232_IND1	1:38,864,179	AGG to A	Unique in mutant	Y
M238	SNP	238_SNP4	8:24,636,417	G to A	Unique in mutant	Y
M238	SNP	238_SNP5	9:19,013,812	G to C	Unique in mutant	Y
M238	indel	238_IND1	1:2,382,470	CG to C	Unique in mutant	Y
M238	indel	238_IND2	1:3,324.566	GGTGGT to G	Unique in mutant	Y
M238	indel	238_IND4	6:12,475,810	C to CAAGT	Unique in mutant	Y
M238	indel	238_IND5	6:16,993,055	TA to A	Unique in mutant	Y
M238	indel	238_IND6	6:17,139,752	A to AGATGCTCTAGGACAGTTTGTTGG	Not unique in Mutant	Y
M242	SNP	242_SNP1	1:14,877,967	G to T	Not unique in mutant	Y
M242	SNP	242_SNP3	3:8,704,769	G to A	Not unique in mutant	Y
M242	SNP	242_SNP5	8:8,951,950	G to T	Unique in mutant	Y
M242	indel	242_IND1	1:13,935,043	A to AT	Low coverage	N
M242	indel	242_IND4	7:29,013,042	CAAGG to C	Unique in mutant	Y
M242	indel	242_IND4	7:29,013,050	G to GC	Unique in mutant	Y
M244	SNP	244_SNP3	7:11,432,559	C to T	Filtered for allele ratio **	N
M244	SNP	244_SNP4	11:22,433,103	C to T	Filtered for allele ratio ***	N
M244	indel	244_IND1	8:24,737,886	A to ACCAACG	Not unique in mutant	Y
M314	SNP	314_SNP2	4:28,342,904	G to T	Unique in mutant	Y
M314	indel	314_IND1	4:3,682,688	G to GAT	Unique in mutant	Y
M314	indel	314_IND1	4:3,682,689	G to GA	Not unique in mutant	Y
M314	indel	314_IND1	4:3,682,697	GGT to G	Unique in mutant	Y
M314	indel	314_IND1	4:3,682,701	CAG to C	Unique in mutant	Y
M314	indel	314_IND2	7:15,991,122	AC to A	Unique in mutant	Y

* 3 of 29 reads supporting alt allele, GATK called as heterozygous. ** 2 of 21 reads supporting alt allele. *** 2 of 25 reads supporting alt allele.

**Table 5:** Locus affected, and function of confirmed SV mutations.

Mutant Line	Mutation Name	Mutation Call	Call Size	Verified Mutation Size	Locus Affected	Function
M149	149_DEL3	7:42,391–44,131	1740	1743 bp	LOC_Os07g01070.1	Membrane transport activity
M153	153_DEL3	2:30,638,481–30,639,067	586	592 bp	Intron after LOC_Os02g50150	
M232	232_DEL3_2	3:15,993,208–15,994,132	924	952 bp	Intron after LOC_Os03g27840	
M242	242_DUP3	5:1,790,511–1,790,511	179	179 bp	LOC_Os05g03972.1	Plant protein, unknown function
M317	317_DEL2	2:30,048,905–30,049,501	596	603 bp	Intron	
M317	317_DEL3	5:86,454–87,418	964	960 bp	Intron region bordering upstream of LOC_Os05g01080	Uncharacterised protein
M166	166_DEL3	3:14,816,703–14,817,352	649	654 bp	Intron	
M225	225_DEL3	3:14,816,703–14,817,352	649	654 bp	Intron	
M193	193_DEL1	2:35,131,943–35,132,357	414	413 bp	Intron after LOC_Os02g57350	
M232	232_DEL1	2:35,131,943–35,132,357	414	413 bp	Intron after LOC_Os02g57350	
M238	238_DEL2	2:35,131,943–35,132,357	414	413 bp	Intron after LOC_Os02g57350	
M242	242_DEL1	2:35,131,943–35,132,357	414	415 bp	Intron after LOC_Os02g57350	
M244	244_DEL1	2:35,131,943–35,132,357	414	413 bp	Intron after LOC_Os02g57350	
M225	225_DEL2	7:606,112–607,134	1022	1000 bp	LOC_Os07g01990.1	Uncharacterised
M193	193_DEL3	7:606,112–607,134	1022	1000 bp	LOC_Os07g01990.1	Uncharacterised
M314	314_DEL2	7:606,112–607,134	1022	1022 bp	LOC_Os07g01990.1	Uncharacterised
M149	149 DEL2	2:34,566,631–34,570,008	3377	Primer pair designed in deletion, WT (1200), MUT (0)	Intron	
M149	149 DEL3	3:34,303,046–34,303,733	687	WT (750), MUT (50)	Intron	
M232	232 DUP3	4:2,243,338–2,244,529	1191	WT (0), MUT (bands at 1200, 550)	LOC_Os04g04660.1	Expressed protein
M238	238 DEL3_2	5:4,851,233–4,852,248	1015	WT (1000), MUT (100)	Intron	
M242	242 DEL2	6:712,560–713,948	1388	WT (400), MUT (200)	Intron	
M242	242 DEL3	10:23,140,818–23,144,340	3522	WT (4200), MUT (450)	LOC_Os10g42910.1, LOC_Os10g42920.1 LOC_Os10g42930.1	Transposon protein, conserved hypothetical protein, expressed protein
M317	317 DEL1_2	7:547,127–550,124	2534	WT (600), MUT (2200), Primer pair designed in deletion, expected WT (609), MUT (0)	LOC_Os07g01904.1	Expressed protein
M244	244 DUP2	3:35,152,931–35,153,261	330	WT (550), MUT (850,550)	LOC_Os03g62040.1	Retrotransposon protein
M232	232 DUP2	3:35,152,931–35,153,261	330	WT (650), (1100,650)	LOC_Os03g62040.1	Retrotransposon protein
M153	153 DEL1_4	10:23,131,290–23,139,022	7732	WT (~8000), MUT (220)	LOC_Os10g42900.1; LOC_Os10g42910.1	Peptide transporter PTR2, transposon protein
M314	314 DEL3_4	10:23,131,290–23,139,022	7732	WT (~8000), MUT (220)	LOC_Os10g42900.1; LOC_Os10g42910.1	Peptide transporter PTR2, transposon protein

**Table 6:** Predicted effect of small variants.

Mutant Line	Missense		Nonsense		Silent		High		Low		Moderate		Modifier	
	Count	%	Count	%	Count	%	Count	%	Count	%	Count	%	Count	%
M149	131	60.93%	1	0.465%	83	38.605%	42	0.261%	91	0.565%	147	0.913%	15,823	98.261%
M153	204	55.135%	3	0.811%	163	44.054%	54	0.286%	209	1.108%	223	1.183%	18,371	97.423%
M166	422	57.415%	4	0.544%	309	42.041%	48	0.129%	374	1.006%	468	1.259%	36,281	97.606%
M193	148	56.705%	1	0.383%	112	42.912%	52	0.253%	141	0.685%	162	0.787%	20,238	98.276%
M225	87	55.769%	0	0.00%	69	44.231%	28	0.221%	82	0.646%	95	0.749%	12,481	98.384%
M232	204	58.621%	4	1.149%	140	40.23%	64	0.273%	164	0.7%	231	0.986%	22,966	98.041%
M238	419	60.201%	4	0.575%	273	39.224%	74	0.173%	353	0.827%	458	1.073%	41,798	97.927%
M242	1363	58.198%	26	1.11%	953	40.692%	264	0.245%	1294	1.203%	1479	1.375%	104,546	97.177%
M244	158	55.052%	5	1.742%	124	43.206%	58	0.287%	159	0.787%	167	0.826%	19,829	98.1%
M314	107	52.709%	2	0.985%	94	46.305%	46	0.363%	107	0.845%	118	0.931%	12,398	97.861%
M317	100	49.751%	4	1.99%	97	48.259%	35	0.292%	111	0.925%	108	0.9%	11,751	97.884%

## Data Availability

Raw sequencing reads have been uploaded to the NCBI database under BioProject ID PRJNA826906 (https://www.ncbi.nlm.nih.gov/bioproject/?term=PRJNA826906, accessed 14 April 2022).

## Supplementary Materials

The following supporting information can be downloaded at: https://www.mdpi.com/article/10.3390/plants11233232/s1. Supplemental Figure S1. Sequencing coverage per chromosome. Supplemental Figure S2. Cross-chromosomal translocations detected in each sample. Supplemental Figure S3. Summary of translocations in all mutants. Supplemental Figure S4. Generation of mutagenic population and selection of candidate mutant lines. Supplemental Table S1. Summary of alignment statistics. Supplemental Table S2. Mean coverage per sample. Supplemental Table S3. Summary of filtering natural point mutations and small indels. Supplemental Table S4. Deletion events called in mutant line M149.
