# Supplementary material for: Spectrum and Density of Gamma and X-ray Induced Mutations in a Non-Model Rice Cultivar
Source: Plants (Basel). 2022 Nov 25;11(23):3232. doi: 10.3390/plants11233232 (PMC9741009; doi:10.3390/plants11233232)
Supplement: Supplementary file 1 [file plants-11-03232-s001.zip › plants-2024997-supplementary.pdf]

Supplemental Figures

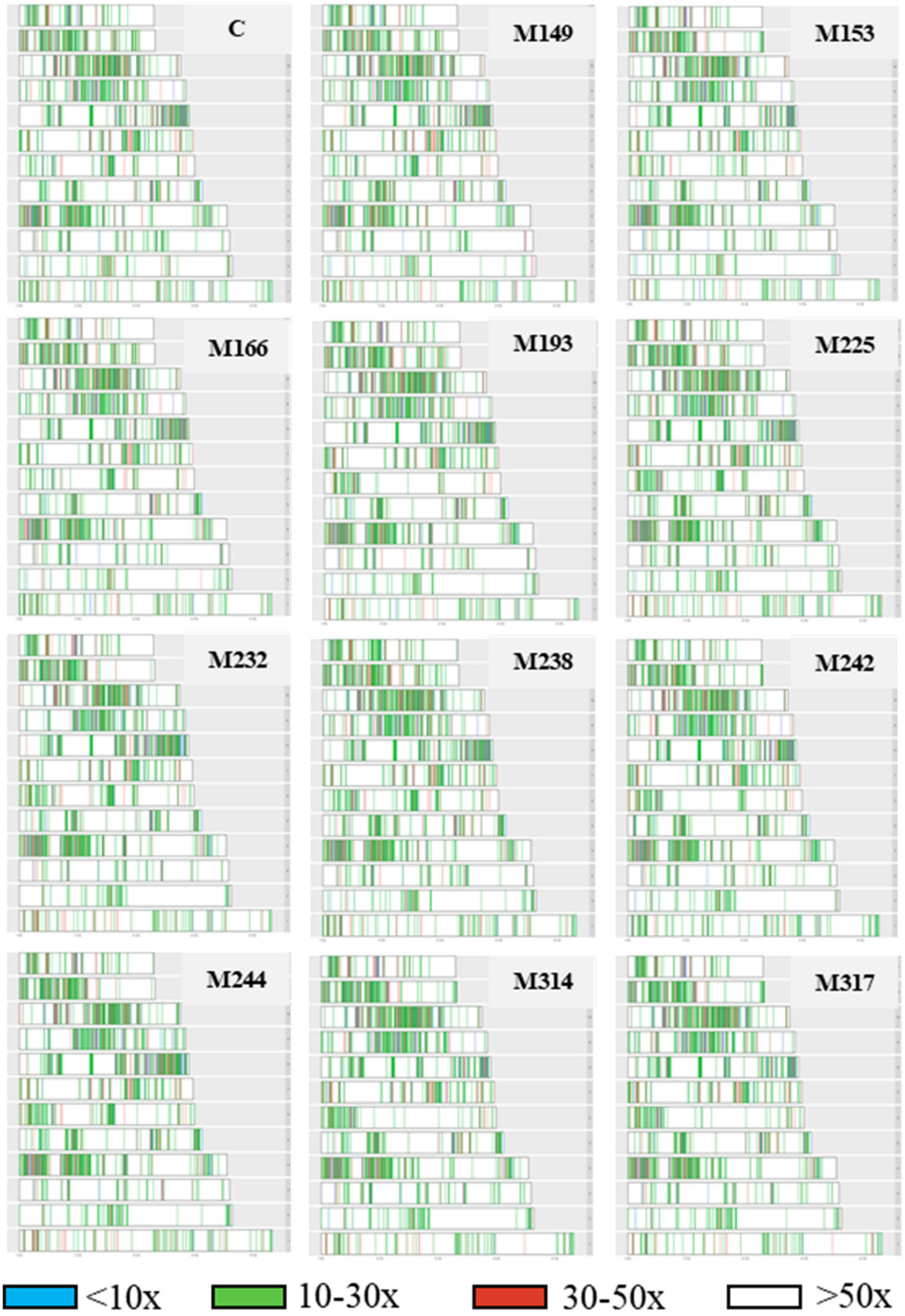

Supplemental Figure S1. Sequencing coverage per chromosome. Coverage of <10x is blue, 10-30x green, 30-50x red and >50x white.

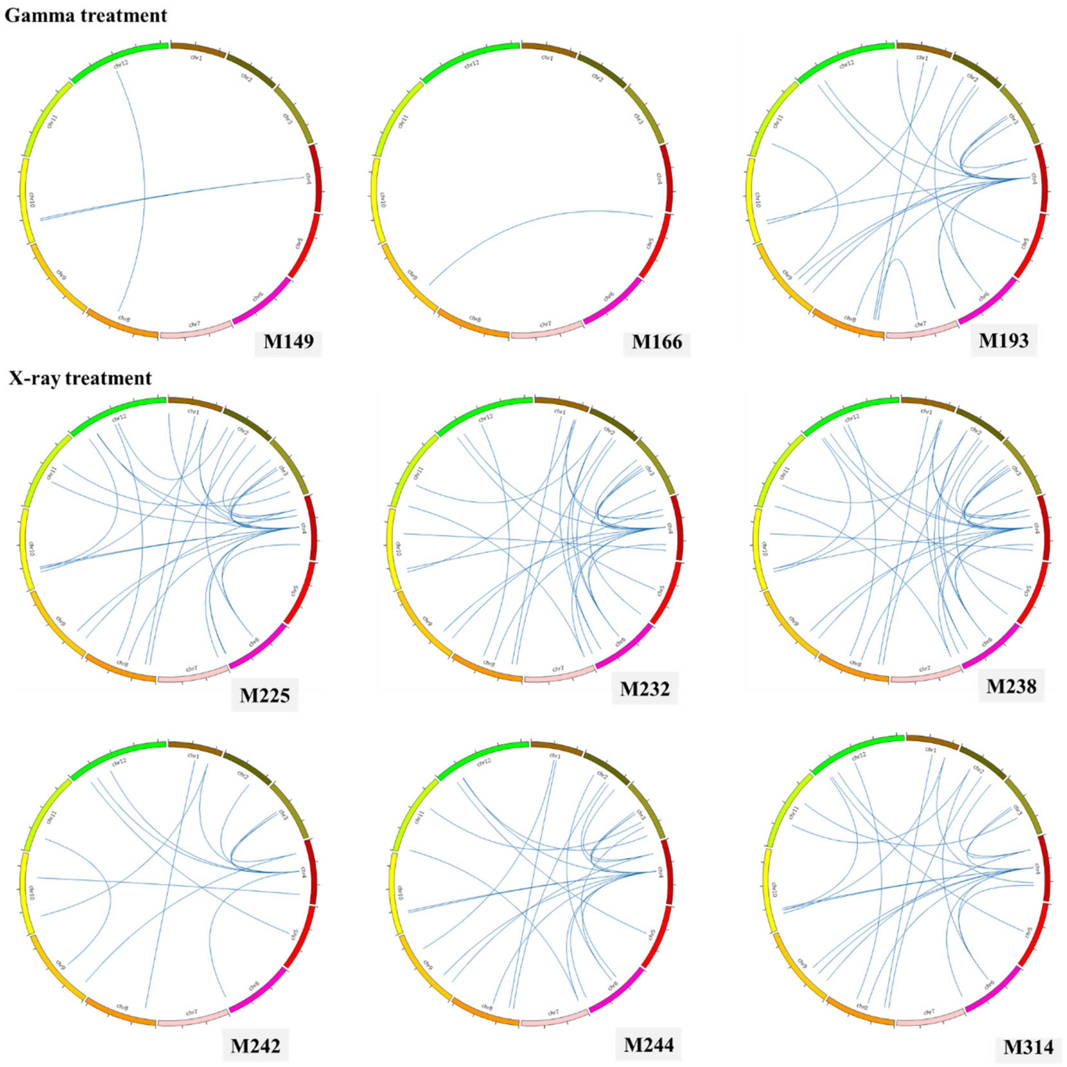

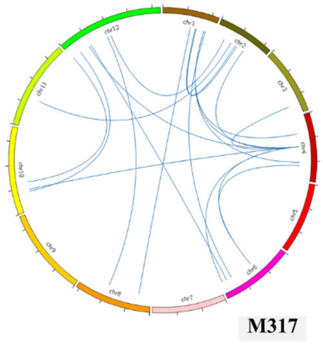

Mutant Line	Treatment type	Dosage
M149	gamma	150 Gy
M153	gamma	150 Gy
M166	gamma	300 Gy
M193	gamma	450 Gy
M225	X-ray	75 Gy
M232	X-ray	76 Gy
M238	X-ray	77 Gy
M242	X-ray	78 Gy
M244	X-ray	79 Gy
M314	X-ray	150 Gy
M317	X-ray	150 Gy

Supplemental Figure S2. Cross-chromosomal translocations detected in each sample. Data from the two replicates were merged together and translocations identified in the control material were removed.

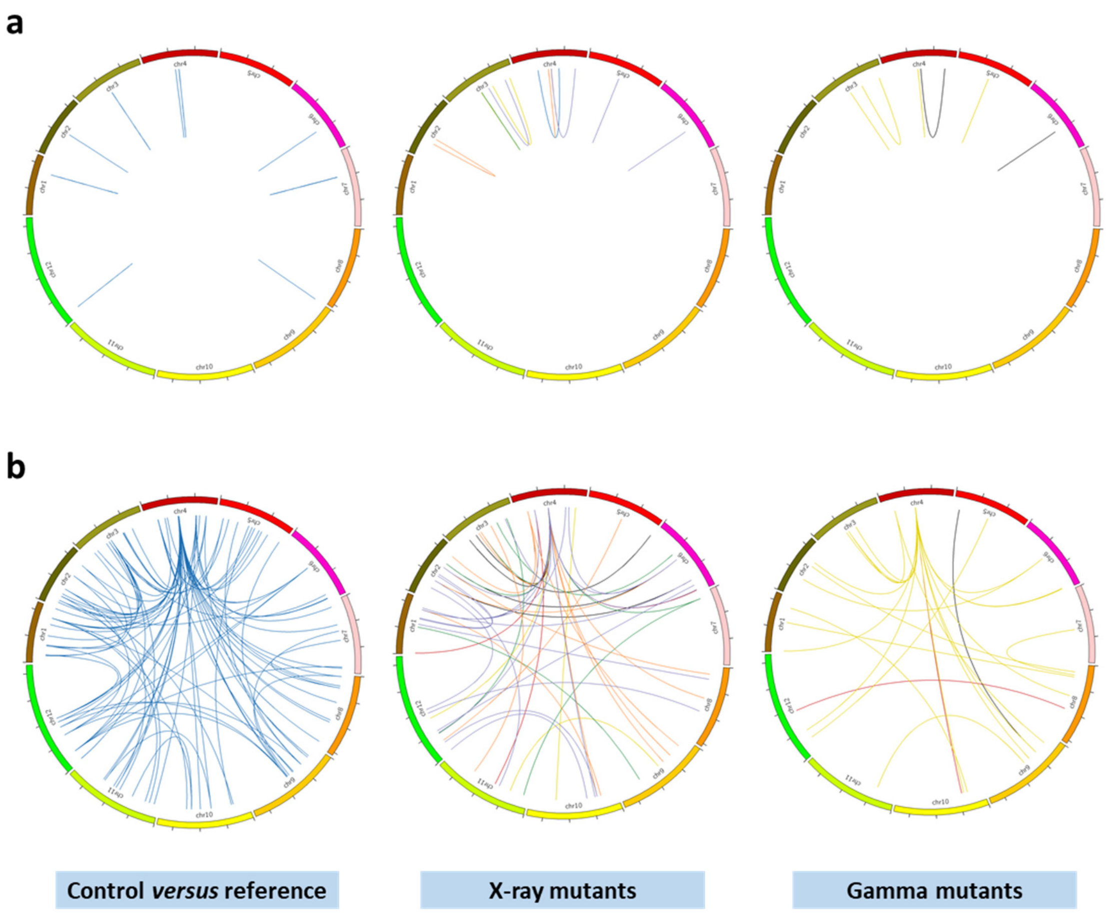

Supplemental Figure S3. Summary of translocations in all mutants. (a) Intrachromosomal translocations identified between control and the reference genome and irradiated material. (b) Cross-chromosomal translocations identified between control and the reference genome and irradiated material. Translocations identified in control material were removed from plots of X-ray and gamma mutants to show only events potentially caused by irradiation.

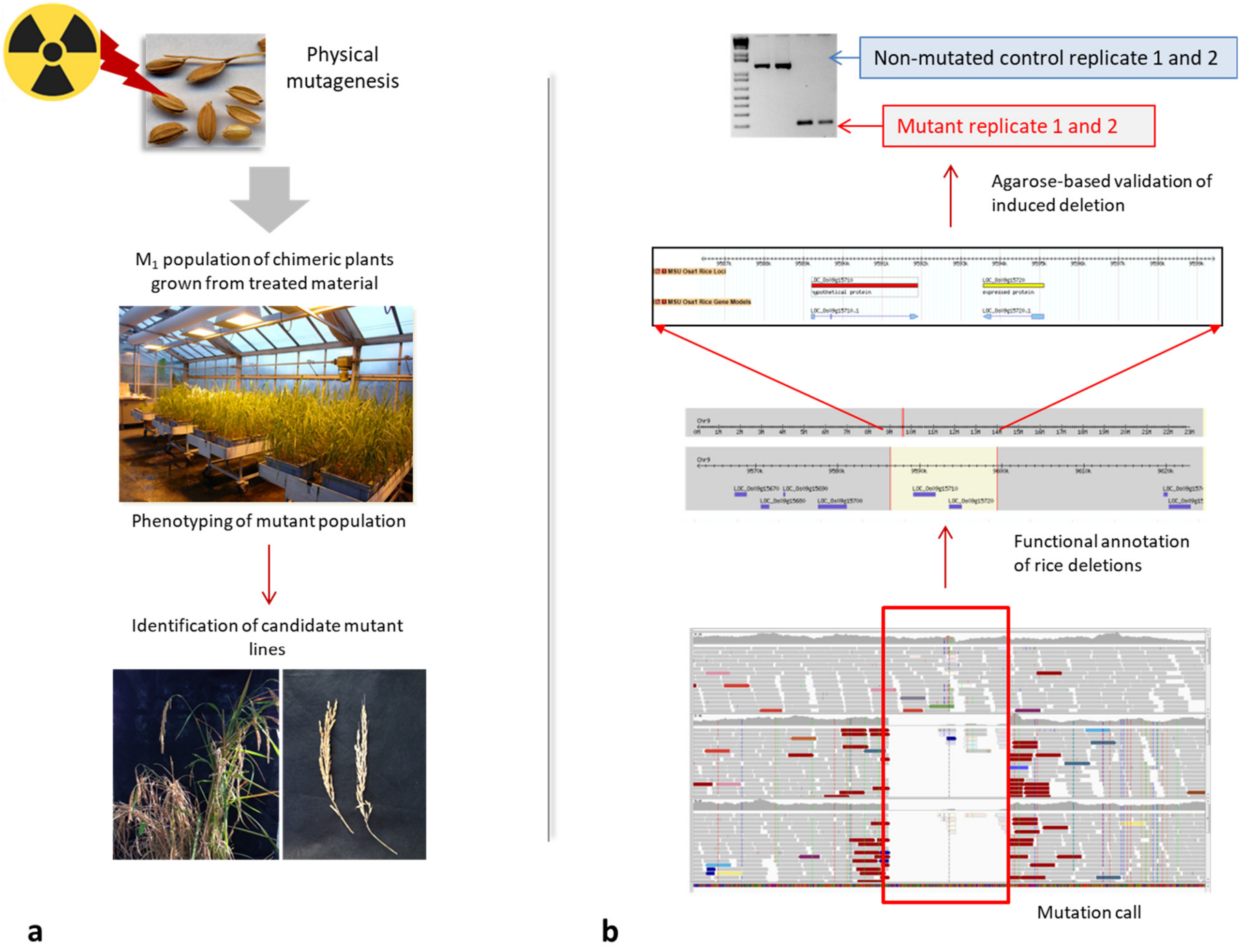

Supplemental Figure S4. Generation of a mutant population and selection of candidate mutant lines. a) X-ray 75 Gy mutant line M242 is being displayed which is characterized by dwarf growth, early maturing, short and dark-brown grain. b) Upon whole genome sequencing, mutations were called, functionally annotated and confirmed *via* Sanger sequencing or gel assay in both biological replicates.
